# Molecular and Phylogenetic Characterization of Novel Papillomaviruses Isolated from Oral and Anogenital Neoplasms of Japanese Macaques (*Macaca fuscata*)

**DOI:** 10.3390/v13040630

**Published:** 2021-04-07

**Authors:** Lucijan Skubic, Lea Hošnjak, Jeannette P. Staheli, Michael R. Dyen, Rebecca M. Ducore, Lois M. A. Colgin, Anne D. Lewis, Mario Poljak

**Affiliations:** 1 Institute of Microbiology and Immunology, Faculty of Medicine, University of Ljubljana, Zaloška 4, 1000 Ljubljana, Slovenia; lucijan.skubic@mf.uni-lj.si (L.S.); lea.hosnjak@mf.uni-lj.si (L.H.); 2 Center for Global Infectious Disease Research, Seattle Children’s Research Institute, 4800 Sand Point Way NE, Seattle, WA 98105, USA; jeannette.staheli@seattlechildrens.org; 3 King County Water and Land Resources Division, Department of Natural Resources and Parks, 322 West Ewing Street, Seattle, WA 98119, USA; mdyen@kingcounty.gov; 4 Oregon National Primate Research Center, Oregon Health & Science University, 505 N.W. 185th Avenue, Beaverton, OR 97006, USA; ducore@ohsu.edu (R.M.D.); colginl@ohsu.edu (L.M.A.C.); lewisann@ohsu.edu (A.D.L.)

**Keywords:** papillomavirus, *Alphapapillomavirus*, *Macaca fuscata*, molecular analysis, phylogenetic analysis, viral load

## Abstract

Papillomaviruses (PVs) are a diverse group of host species-specific DNA viruses, etiologically linked with various benign and malignant neoplasms of cutaneous and mucosal epithelia. Here, we describe the detection and characterization of the first two PVs naturally infecting Japanese macaques (*Macaca fuscata*), including the determination of their etiological association(s) with the development of original neoplasms. The molecular and phylogenetic analyses were performed on complete genome sequences of Macaca fuscata PV types 1 (MfuPV1) and 2 (MfuPV2), which were completely sequenced in samples of a malignant oral tumor and benign anogenital neoplasm of Japanese macaques, respectively. Subsequently, two type-specific quantitative real-time PCRs were developed to estimate viral loads of MfuPV1 and MfuPV2 and to evaluate their etiological roles. The in silico molecular analyses revealed that both viral genomes encode characteristic PV proteins with conserved functional domains and have a non-coding genomic region with regulatory sequences to regulate and complete the viral life cycle. However, additional experimental evidence is needed to finally confirm the presence and biological functionality of the molecular features of both novel PVs. While MfuPV1, together with PVs identified in other macaques, is classified into the *Alphapapillomavirus* (*Alpha*-PV) species 12, MfuPV2 is most likely a representative of the novel viral species within the *Alpha*-PV genus. Their relatively high viral loads suggest that both PVs are etiologically linked with the development of the original neoplasms.

## 1. Introduction

Papillomaviruses (PVs) are a large and diverse family of host species-specific, small, non-enveloped DNA viruses that infect cutaneous and mucosal stratified epithelia in a wide range of vertebrates, where they can cause persistent asymptomatic infections and the development of various benign and malignant neoplasms [1,2]. PVs have circular, double-stranded DNA genomes with sizes close to 8 kilobase pairs (kb), which typically consist of seven or eight distinct open reading frames (ORFs), encoding viral proteins, and a non-coding long control region (LCR) with numerous regulatory elements that regulate viral transcription and replication [3]. Based on the nucleotide similarity of the highly conserved full-length L1 ORFs, PVs are hierarchically classified into genera, species, and types. PV types are etiologically linked to specific diseases, and those within the same viral species typically have very similar biological or pathological properties (e.g., tissue tropism and tumorigenic potential). A novel PV type is established when its full-length genome has been cloned into a plasmid vector and its L1 ORF nucleotide sequence differs from that of any other already characterized and officially recognized PV type by at least 10%. With a few exceptions, each PV type is named sequentially using the scientific name and its abbreviation of the original host species and the serial number of the isolate (e.g., Pan paniscus papillomavirus type 1—PpPV1). PV types that share 60–70% L1 sequence similarity belong to different PV species, and those with similarities less than 60% are members of different viral genera [1,4,5]. As of 26 February, 2021, 437 PV types, identified from 92 different host species, have been officially recognized and classified into 49 separate PV genera [6].

Most of the currently recognized PVs have been identified in primates, including 222 human PV (HPV) and 28 non-human primate (NHP) PV types, which are classified into six viral genera: *Alphapapillomavirus* (*Alpha*-PV), *Betapapillomavirus* (*Beta*-PV), *Gammapapillomavirus* (*Gamma*-PV), *Mupapillomavirus* (*Mu*-PV), *Nupapillomavirus* (*Nu*-PV), and *Dyoomikronpapillomavirus* (*Dyoomikron*-PV) [6]. NHP PV types have been detected in a variety of monkeys and apes (Simiiformes), including brown howlers (*Alouatta guariba*), colobus monkeys (*Colobus guereza*), common squirrel monkeys (*Saimiri sciureus*), cynomolgus macaques (*Macaca fascicularis*), rhesus macaques (*Macaca mulatta*), hamadryas baboons (*Papio hamadryas*), and pygmy chimpanzees (*Pan paniscus*). Because of the similarities between humans and NHPs and the long history of co-evolution of PVs with their hosts, NHP PVs are phylogenetically most closely related to HPVs. This makes the study of NHP PVs even more important, as they can potentially be used as model systems in the pathogenesis studies of HPV-related diseases [7,8]. The best-studied NHP PVs to date (Macaca fascicularis PV (MfPV) types 1–11 and Macaca mulatta PV (MmPV) types 1–7) have been identified in cynomolgus and rhesus macaques, species belonging to the same genus as Japanese macaques (*Macaca fuscata*) (Table 1) [9,10]. While MfPV1 and MfPV2, identified in palmoplantar skin papillomas, are members of the *Beta*-PV genus [8,10], other PVs of macaques originate from anogenital mucosa and are classified as *Alpha*- (MfPV3–11, MmPV1–3 and MmPV6) and *Gamma*-PVs (MmPV4, MmPV5 and MmPV7) [11,12]. Specifically, MfPV3–11 were originally identified in exfoliated cervical cytology samples of cynomolgus macaques, with MfPV3, MfPV4, MfPV5, and MfPV8 being associated with the development of cervical intraepithelial neoplasia. Additionally, while MmPV1 was originally detected in a lymph node metastasis of a penile squamous cell carcinoma of a rhesus macaque [11,13], MmPV2–7 were identified in exfoliated vaginal cells and swabs of penile surfaces of rhesus macaques [12].

In the present study, we describe the detection and characterization of the first PV types naturally infecting Japanese macaques. We detected Macaca fuscata papillomavirus types 1 (MfuPV1) and 2 (MfuPV2) in oral and anogenital neoplasms of Japanese macaques, respectively, using broad-spectrum CODEHOP PCR, followed by full genomic sequencing and a detailed molecular characterization and phylogenetic evaluation. We subsequently developed two type-specific quantitative real-time PCRs (qPCRs) and used them in combination with the tubulin qPCR to estimate the MfuPV1 and MfuPV2 viral loads and to evaluate their etiological role in the development of the original neoplasms.

## 2. Materials and Methods

### 2.1. Tumor Specimens

Fresh tissue samples of oral and penile lesions were obtained at necropsy of the Japanese macaques at the Oregon National Primate Research Center (Oregon Health & Science University, Beaverton, OR, USA) in 2014–2015 and divided into two parts; one part of each sample was stored at −80 °C for DNA extraction and downstream PCR analyses, and the second part was fixed with formalin and embedded into paraffin for histopathological analysis.

**Figure 1:**
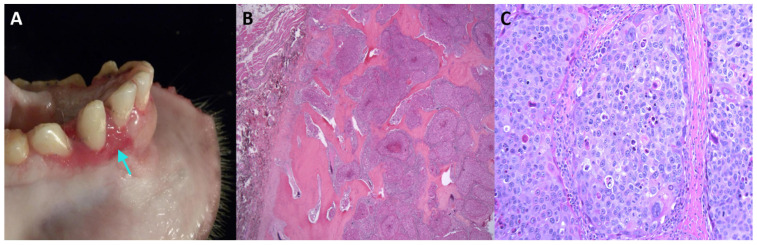
Oral squamous cell carcinoma in an adult female Japanese macaque. (**A**) Gross pathology of the oral lesion with a focus of gingival elevation and erosion on the rostral mandible (indicated by arrow). (**B**) Histopathological section showing a multilobulated, infiltrative neoplastic mass invading and effacing the mandibular bone (stained with hematoxylin and eosin, 20× magnification). (**C**) Higher magnification of (**B**) showing neoplastic cells arranged in islands (200× magnification). Neoplastic cells are polygonal, with distinct cell borders, a moderate amount of eosinophilic cytoplasm, large central nuclei with marked karyomegaly, multifocal multinucleated cells, and prominent nucleoli. Scattered individual neoplastic cells are shrunken and necrotic.

We detected MfuPV1 in an oral lesion of a 14-year-old female Japanese macaque (Figure 1). Specifically, the mentioned lesion was present as a fleshy, pink, soft tissue mass associated with the buccal gingiva of the mandibular incisors and extended into the buccal mucosa, lingual gingiva, and base of the frenulum. Histopathological analysis showed that the oral mucosa was ulcerated, and the submucosa was expanded by a poorly demarcated, densely cellular, endophytic and exophytic population of neoplastic epithelial cells arranged in nests on a densely collagenous to scirrhous stroma. Similar nests of neoplastic cells infiltrated and effaced the mandibular symphysis cortical and trabecular bone. Rafts of neoplastic cells were present within the lumen of salivary gland ducts in one section of the lingual mucosal surface and within the subcapsular sinuses of both submandibular lymph nodes. Finally, in the described tissue, pathologic findings confirmed the presence of a locally aggressive oral squamous cell carcinoma with mandibular invasion and submandibular lymph node metastasis (Table 1).

**Figure 2:**
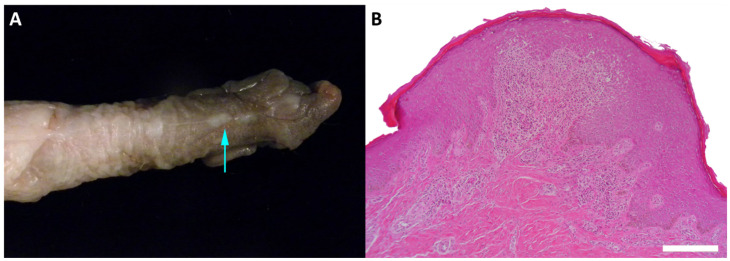
Benign penile lesion in a juvenile Japanese macaque. (**A**) Gross appearance of the irregular, white minimally raised epithelial nodule on the surface of the penis (indicated by arrow). (**B**) Histopathological section showing an epithelial hyperplasia with downgrowth of epithelial cords and dermal inflammation (stained with hematoxylin and eosin, 100× magnification).

We identified MfuPV2 in a benign penile lesion of a 4-year-old Japanese macaque (Table 1, Figure 2). Irregular, white minimally raised epithelial nodules that measured up to 0.2 cm in greatest dimension were present over the glans and corpus of the penis. Histopathological examination showed that microscopic features of the nodules included mild epithelial hyperplasia with downgrowth of epithelial cords, which is consistent with papillomas.

The animals were maintained in research colonies at the Oregon National Primate Research Center, which is fully accredited by the Association for Assessment and Accreditation of Laboratory Animal Care International. Animal care and procedures were approved by the Institutional Animal Care and Use Committee and were conducted to ensure compliance with the U.S. Animal Welfare Act, Guide for the Care and Use of Laboratory Animals [14], and other prevailing statutes, regulations and guidelines.

### 2.2. DNA Extraction and Amplification of Partial Viral Sequences

Total DNA from fresh-frozen samples of the original neoplasms, containing MfuPV1 or MfuPV2, was extracted by an overnight digestion with proteinase K (Qiagen, Hilden, Germany) at 56 °C, followed by DNA purification with a QIAamp DNA Mini Kit (Qiagen), according to the manufacturer’s protocol for tissue DNA extraction. Subsequently, total DNA was eluted in 200 µL of TE buffer (10 mM Tris-Cl, 0.5 mM EDTA; pH 9.0), and 5 µL was used for the CODEHOP PCR reaction.

Partial viral nucleotide sequences (ca. 400 base pairs (bp)) of MfuPV1 and MfuPV2 L1 ORFs, suggesting the presence of putative novel representatives of the *Alpha*-PV genus, were initially obtained using broad-spectrum pan-PV CODEHOP primers, following a single-tube nested “hanging droplet” PCR protocol, as described previously [15]. Amplicons were gel-purified via a 2% agarose gel using a QIAquick Gel Extraction Kit (Qiagen) and sequenced by Sanger sequencing. Subsequently, phylogenetic analysis of the mentioned sequences was performed by the maximum likelihood method (PhyML as part of the Geneious v6.1.6 software package; Biomatters Ltd., Auckland, New Zealand), using known primate (human and NHP) PV types with fully recognized genomes. Searches of the GenBank database (NCBI, U.S. National Library of Medicine, Bethesda, MD, USA) were additionally performed using the NCBI Nucleotide BLAST web-based service (https://blast.ncbi.nlm.nih.gov/Blast.cgi, accessed on 21 November 2016) to determine the most closely related PV types.

Total DNA extracted from the penile lesion was additionally subjected to rolling circle amplification (RCA) using an Illustra TempliPhi 100 Amplification Kit (GE Healthcare Life Sciences, Little Chalfont, UK), as described previously [16]. The resulting RCA product was diluted in nuclease free water (ratio 1:100) and used in downstream PCR analyses.

### 2.3. Amplification of the Complete MfuPV1 and MfuPV2 Genomes

The MfuPV1-specific primer set (MfuPV-F3: 5’-GATGCCATCTTTAGGTAATCTGGA-3’ and MfuPV-R3: 5’-AGAGCCCTATGGTGACAGTATGTT-3’), targeting the MfuPV1 L1 genomic region, and two MfuPV2-specific primer sets (MfuPV2-249f1: 5’-CAATCAAGGGGACACAGTCC-3’ and MfuPV2-145r1: 5’-TGCTCACGCCTCAGATAAAA-3’; MfuPV2-E1-invforward: 5’-GGACACAGTGCTGAAAACTCT-3’ and MfuPV2-E1-invreverse: 5’-CTGTTCTTCGCACATTTGAA-3’), targeting the MfuPV2 L1 and E1 genomic regions, respectively, were designed and evaluated using the Primer3 v0.4.0 (http://bioinfo.ut.ee/primer3-0.4.0/, accessed on 14 March 2017) and Net Primer (http://www.premierbiosoft.com/netprimer/, accessed on 14 March 2017) web-based applications. To avoid potential cross-reactivity with non-targeted sequences, the specificity of the designed primers was additionally verified using the NCBI Nucleotide BLAST web-based service. The type-specific primer sets were subsequently used to amplify the complete viral genomes using inverted long-range PCRs. While the use of the MfuPV1-specific primer set enabled the MfuPV1 genome to be amplified along its entire length, the full-length MfuPV2 genome was amplified by the combination of MfuPV2-specific primer sets, respectively, generating two types of amplicons of approximately 4 kb, corresponding to two genomic halves of MfuPV2 DNA.

All type-specific inverted long-range PCRs were conducted using a Platinum *Taq* DNA Polymerase High Fidelity Kit (Invitrogen, Carlsbad, CA, USA) in a 25 μL reaction mixture containing 5 µL of sample DNA or diluted RCA product, 2.5 µL of 10× High Fidelity PCR Buffer, 1 µL of 50 mM MgSO_4_, 200 µM of dNTPs (Roche, Mannheim, Germany), 0.2 µM of each primer, 0.5 U of Platinum *Taq* High Fidelity DNA polymerase, and nuclease free water. The MfuPV1-specific inverted long-range PCR protocol was performed on a Veriti Thermal Cycler (Applied Biosystems, Foster City, CA, USA) under the following conditions: initial DNA denaturation for 2 min at 94 °C, followed by 45 amplification cycles of 30 s at 94 °C, 30 s at 58 °C, and 8 min at 68 °C. In the case of using MfuPV2-specific primer sets, the cycling conditions were as follows: initial DNA denaturation for 2 min at 94 °C, followed by 45 amplification cycles of 30 s at 94 °C, 30 s at 56 °C, and 4 min at 68 °C. The final elongation step in all PCRs was performed at 68 °C for 7 min, followed by cooling of the reaction mixture to 8 °C.

### 2.4. Cloning of the Complete MfuPV1 and MfuPV2 Genomes

The obtained PCR products were separated by a 0.8% agarose gel electrophoresis dyed with a fluorescent SYBR Safe DNA gel stain (Invitrogen). In the case of MfuPV1, visible bands of the expected size of 8 kb were purified from the gel using a QIAquick Gel Extraction Kit (Qiagen) and cloned into pCR-XL-TOPO plasmid vectors with a TOPO XL PCR Cloning Kit (Invitrogen), following the manufacturer’s instructions. In contrast, both types of MfuPV2 PCR products, each with an expected size of 4 kb, were purified using a QIAquick PCR Purification Kit (Qiagen) and cloned into pJET1.2/blunt plasmid vectors using a CloneJET PCR Cloning Kit (Thermo Scientific, Waltham, MA, USA), as instructed by the manufacturer. The recombinant plasmids were subsequently transformed into One Shot TOP10 Chemically Competent *Escherichia coli* cells (Invitrogen), according to the manufacturer’s instructions. The transformants were incubated for 16 h at 37 °C on Luria–Bertani agar plates with kanamycin (50 μg/mL) or ampicillin (50 μg/mL), respectively, depending on the plasmid vectors used for the molecular cloning process. Bacterial colonies containing plasmids with inserts of the appropriate size were detected by colony PCR using a Platinum *Taq* DNA Polymerase High Fidelity Kit (Invitrogen) in combination with M13 Forward/M13 Reverse primers (TOPO XL PCR Cloning Kit, Invitrogen) or pJET1.2 Forward/pJET1.2 Reverse primers (CloneJET PCR Cloning Kit, Thermo Scientific), respectively, following the manufacturers’ instructions, on a Veriti 96-Well Thermal Cycler (Applied Biosystems). Plasmid DNA of clones harboring appropriate PV inserts was extracted from 4 mL of bacterial culture, grown for 16 h at 37 °C in a Luria–Bertani liquid medium supplemented with 50 μg/mL kanamycin or 50 μg/mL ampicillin, respectively, using a QIAprep Spin Miniprep Kit (Qiagen), as instructed by the manufacturer. The extracted DNA was quantified by a NanoDrop 2000c spectrophotometer (Thermo Scientific).

### 2.5. Sequencing of the Complete MfuPV1 and MfuPV2 Genomes

The cloned MfuPV1 PCR products were sequenced on both strands using a primer-walking strategy at Microsynth AG (Balgach, Switzerland). The primers used for MfuPV1 whole genome sequencing are listed in Table S1.

Sanger sequencing reactions of both types of cloned MfuPV2 PCR products were performed by a primer-walking strategy using newly designed sequencing primers (Table S2) and a BigDye Terminator v3.1 Cycler Sequencing Kit (Applied BioSystems) on a Veriti 96-Well Thermal Cycler (Applied Biosystems), as described previously [17]. Products of sequencing reactions were subsequently purified using a DyeEx 2.0 Spin Kit (Qiagen), according to the manufacturer’s instructions, and analyzed on an automated sequencing instrument ABI3500 Genetic Analyzer (Applied Biosystems). The obtained electropherograms were initially inspected with the Sequencing Analysis Software v5.4 package (Applied Biosystems).

### 2.6. Molecular Analysis of MfuPV1 and MfuPV2

Complete nucleotide sequences of MfuPV1 and MfuPV2 genomes were assembled and edited using the Vector NTI Advance v11.5.4 software package (Invitrogen). PV-specific ORFs were determined using the ORF finder web-based service (https://www.ncbi.nlm.nih.gov/orffinder/, accessed on 10 September 2017). Published literature and several freely available web-based applications, including GPMiner [18], SIGSCAN software v4.05 [19], Patch 1.0-Gene Regulation (http://gene-regulation.com/pub/programs.html#patch, accessed on 10 September 2017), Poly(A) Signal Miner [20], and 2ZIP-Server [21], were additionally used to characterize viral genomic regions and functional protein domains/motifs, as described previously [22,23]. Detailed molecular characterization was performed by comparing nucleotide and amino acid alignments of individual genomic regions and protein sequences of MfuPV1, MfuPV2, and their closest relatives, using the MAFFT v7.402 algorithm [24] and the BioEdit Sequence Alignment Editor v7.2.6.1 software package (Ibis Therapeutics, Carlsbad, CA, USA).

### 2.7. Phylogenetic Analysis of MfuPV1 and MfuPV2 and Sequence Similarities

In order to phylogenetically place MfuPV1 and MfuPV2 within the *Papillomaviridae* family, a multiple sequence alignment of the full-length L1 ORF nucleotide sequences of MfuPV1, MfuPV2, and 142 previously characterized and officially recognized PV types from the *Alpha*- (*n* = 80), *Beta*- (*n* = 50), *Gamma*- (*n* = 6), *Mu*- (*n* = 3), and *Dyoomikron*-PV (*n* = 3) genera was constructed using the MAFFT v7.402 algorithm [24]. All L1 ORF sequences of previously characterized PV types were obtained from the Papillomavirus Episteme (PaVE) database [6], which is linked to the GenBank database (NCBI). A maximum likelihood phylogenetic tree with a 1000 bootstrap resampling of the alignment data sets with the GTRCAT evolutionary model for the bootstrapping phase was constructed using the RAxML-HPC2 v8.2.10 algorithm [25]. The phylogenetic tree was visualized and acquired with the MEGA7.0.26 software package (Institute for Genomics and Evolutionary Medicine, Temple University, Philadelphia, PA, USA) [26]. Nucleotide sequences of HPVs from the *Mu*-PV genus were used to root the tree.

In order to compare the genomes of MfuPV1 and MfuPV2 with the genomes of five most closely related PV types from the *Alpha*-PV genus, additional pairwise nucleotide and amino acid sequence alignments were performed. Percentage similarities for each of the MfuPV1/MfuPV2 viral ORFs and proteins and the most related PV types were calculated using the BioEdit Sequence Alignment Editor v7.2.6.1 software package (Ibis Therapeutics). The NCBI PASC—Pairwise Sequence Comparison web-based service [27] was additionally used to calculate MfuPV1 and MfuPV2 whole genome pairwise similarities with all previously characterized PV types.

The GenBank (NCBI) accession numbers of PV types included in the phylogenetic and sequence similarity analyses are listed in Table S3.

### 2.8. MfuPV1 and MfuPV2 Type-Specific Quantitative Real-Time PCR Assays

The MfuPV1-specific qPCR primer set (MfuPV1-E1-1forward: 5’-AAAAGCAACGCACAAGCAAAA-3’ and MfuPV1-E1-1reverse: 5’-TTTGTCTCCAGTCCCCACCTT-3’), targeting the 145 bp fragment of the MfuPV1 E1 genomic region, and the MfuPV2-specific qPCR primer set (MfuPV2-E1-RTforward: 5’-TGATGAGGATGAGGAGGAGGAC-3’; MfuPV2-E1-RTreverse: 5’-ATCTGCTTTCGCTTCTTGCTG-3’), targeting the 133 bp fragment of the MfuPV2 E1 genomic region, were designed and evaluated in the same way as inverted long-range PCR primer sets.

To determine the presence of MfuPV1 and MfuPV2 in tissues, type-specific qPCR was performed using the QuantiTect SYBR Green PCR + UNG Kit (Qiagen) in a 25 µL reaction mixture, consisting of 1–5 μL of template DNA (up to 1000 ng), 12.5 µL 2× QuantiTect SYBR Green PCR Master Mix, 0.5 μM of each primer, and nuclease free water. Both qPCR reactions targeting MfuPV1 or MfuPV2 were conducted on a LightCycler 2.0 Instrument (Roche) under the same conditions, as follows: initial DNA denaturation for 15 min at 95 °C (temperature transition rate 20 °C/s), followed by 45 amplification cycles of 15 s at 94 °C (20 °C/s), 20 s at 60 °C (20 °C/s), and 20 s at 72 °C (2 °C/s). Acquisition of the fluorescence signal (530 nm) was performed in a single mode at the end of the elongation step of each amplification cycle. To verify the specificity of MfuPV1 and MfuPV2 amplicons, a melting curve analysis consisting of three temperature steps—0 s at 95 °C (20 °C/s) and 30 s at 50 °C (20 °C/s), followed by 0 s at 95 °C at a temperature transition rate of 0.1 °C/s (continuous monitoring of the fluorescence)—was performed following the amplification. The final step consisted of cooling of the reaction mixture to 40 °C with a 30 s hold (20 °C/s).

Samples were considered MfuPV1- or MfuPV2-positive when showing specific melting peaks at 79.9 °C or 81.4 °C, respectively. To evaluate the linearity of the MfuPV1 and MfuPV2 qPCR assays, 10-fold serial dilutions of MfuPV1 and MfuPV2 reference plasmids were prepared in 1.5 mL DNA LoBind tubes (Eppendorf, Hamburg, Germany) using a water solution with carrier RNA (1 µg/mL) (Qiagen). The analytical sensitivity of each qPCR assay was determined as described previously [28] by testing triplicates of reference plasmid serial dilutions corresponding to an input of 1 × 10^8^ to 1 × 10^−1^ copies of plasmid DNA per reaction. The detection limit of both the MfuPV1 and MfuPV2 qPCR assay was established at 10 viral copies per reaction. Dynamic ranges of both qPCRs were 7 orders of magnitude and enabled reliable quantification of 10^8^ to 10 viral copies per reaction. The calculated coefficients of determination of MfuPV1 and MfuPV2 qPCR standard curves were 0.999 and 0.998, respectively. MfuPV1 and MfuPV2 qPCR amplification efficiencies were estimated at 89.1% and 98.6%, respectively.

### 2.9. MfuPV1 and MfuPV2 Viral Load Calculation

To determine average cellular viral loads of both novel PVs in the original neoplasms, the MfuPV1 and MfuPV2 qPCR assays were used in combination with a qPCR assay targeting the cellular host gene tubulin, as described previously [23]. In all calculations it was assumed that one Japanese macaque diploid cell contained 7.12 pg of genomic DNA [29].

### 2.10. Data Availability

The complete genome sequences of MfuPV1 (isolate OSCC1) and MfuPV2 (isolate 14A-881) were deposited in the GenBank database (NCBI) under the accession numbers KT944080 and MH469677, respectively.

## 3. Results

### 3.1. Genomic Organization of MfuPV1 and MfuPV2

**Figure 3:**
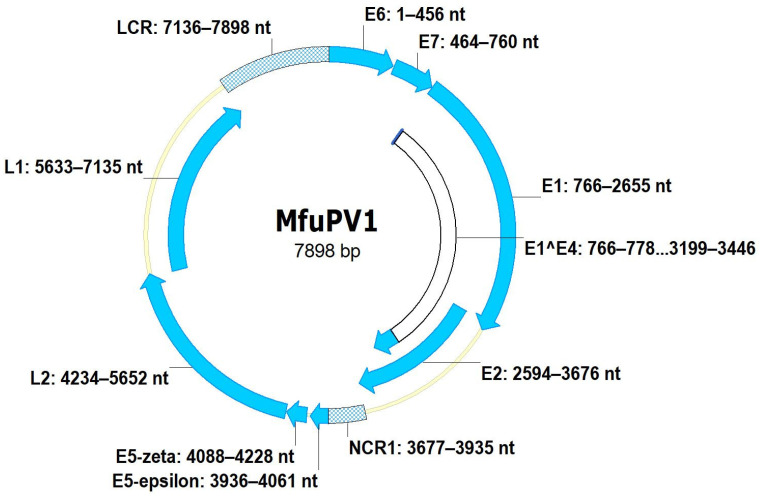
Genomic organization of MfuPV1. Genomic positions of viral open reading frames (E1, E2, E4 (E1^E4), E5-epsilon, E5-zeta, E6, E7, L1, and L2) and non-coding regions (long control region (LCR) and non-coding region 1 (NCR1)) are indicated next to the respective genomic regions.

**Figure 4:**
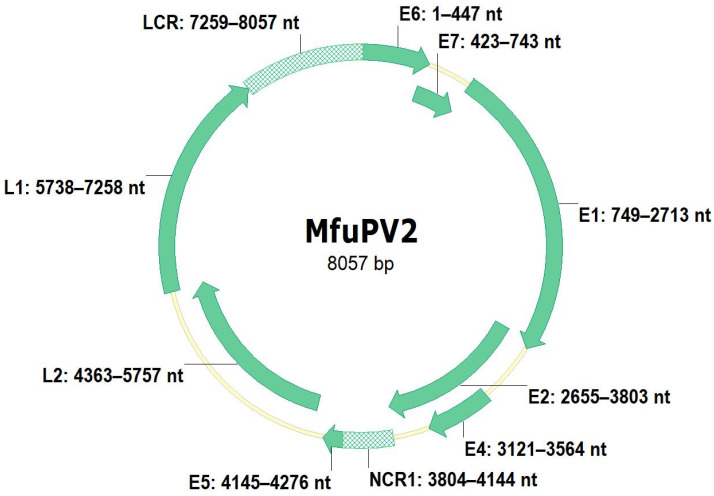
Genomic organization of MfuPV2. Genomic positions of viral open reading frames (E1, E2, E4, E5, E6, E7, L1, and L2) and non-coding regions (long control region (LCR) and non-coding region 1 (NCR1)) are indicated next to the respective genomic regions.

The complete nucleotide sequence of the MfuPV1 genome is 7898 bp in length, with a GC content of 47.1%, while the complete MfuPV2 genome is 8057 bp in length and has a GC content of 50.8%. As shown in Figure 3 and Figure 4, both novel viral genomes contain eight major open reading frames (ORFs), encoding six early (E1, E2, E4, E5, E6, and E7) and two late viral proteins (L1 and L2), where MfuPV1 encodes two different E5 proteins, E5-epsilon and E5-zeta. In addition, there is a 763 and 799 bp LCR positioned between the L1 and E6 ORFs of the MfuPV1 and MfuPV2 genomes, respectively. The LCRs of both viral genomes contain putative polyadenylation sites (AATAAA) for processing late viral mRNA transcripts at their 5’ ends, where in MfuPV2 an additional polyadenylation site (ATTAAA) was observed (Table 2). Furthermore, the LCRs of both novel PVs harbor four consensus palindromic E2-binding sites (ACC(N)_6_GGT); however, the fourth MfuPV2 E2-binding site is slightly modified (ACC(N)_6_GCT). Both LCRs also contain an 18 bp long palindromic E1-binding region consisting of six overlapping recognition sequences for the E1 protein with a consensus AACNAT or AT(A/G/T)G(C/T)(C/T) sequence. A putative TATA box motif (TATAAA) of the major early viral promoter is located at the 3’ ends of the MfuPV1 and MfuPV2 LCRs. In addition, numerous putative binding sites for cellular transcription factors, including C/EBPbeta, YY1, AP-1, NFI/CTF, and SP-1, were identified in the LCRs of MfuPV1 and MfuPV2. Both viral genomes contain additional non-coding regions (NCR1s) located between the E2 and E5 ORFs (Figure 3 and Figure 4). A putative polyadenylation site (AATAAA) for processing early viral mRNA transcripts is located at the 3’ end of the MfuPV1 E5-zeta ORF and next to the 5’ end of the MfuPV2 L2 ORF (Table 2).

### 3.2. Characteristics of MfuPV1 and MfuPV2 Proteins

The E1 ORFs encode the largest MfuPV1 (629 amino acid residues) and MfuPV2 (654 amino acid residues) proteins. Both contain a bipartite nuclear localization signal (NLS), comprised of two regions of arginine and lysine residues (basic, positively charged amino acids), separated by 32 amino acids, and a leucine-rich nuclear export signal (NES) (Lx_(2–3)_Lx_2_(L/I/V)x(L/I)), located between the two basic NLS regions within the amino (N-) terminal region (Table 2). Interestingly, the downstream NLS region of the MfuPV1 E1 protein (SQAK) consists of nonpolar and polar uncharged amino acids. The N-terminal regions of both MfuPV1 and MfuPV2 E1 proteins also contain several putative phosphorylation sites ((S/T)P) for the cyclin-dependent kinase 2 (Cdk2) and a consensus cyclin-binding motif (RxL). Furthermore, a conserved ATP-binding site with a consensus Gx_4_GK(T/S) sequence was identified at the carboxy (C-) termini of both E1 proteins. Both novel PVs have a consensus NLS (KRxR) identified in the central hinge regions and a conserved leucine-zipper domain (Lx_6_Lx_6_Lx_6_L) at the C-termini of E2 proteins.

Both E4 ORFs are located centrally within the E2 ORFs (Figure 3 and Figure 4). While the MfuPV2 E4 ORF contains a start codon, the MfuPV1 E4 ORF lacks one. However, the identification of characteristic donor (AAG/GUASNR) and acceptor (GUYACYAG/YU) RNA splicing sites suggests that E4 proteins of both novel PVs can be translated as fusion proteins from spliced E1^E4 mRNAs. A consensus “leucine-cluster” motif (LLxLL) was identified at the N-terminus of the MfuPV1 E4 protein, whereas MfuPV2 harbors a L64V substitution on the fifth position of the motif (Table 2). The putative MfuPV1 E1^E4 fusion protein and MfuPV2 E4 protein contain 11.6% (10/86 amino acid residues) and 9.5% (14/147 amino acid residues) proline residues, respectively. As previously mentioned, the MfuPV2 genome has one E5 ORF, positioned between E2 and L2 ORFs, while the MfuPV1 genome contains two E5 ORFs, located in the region between E2 and L2 ORFs. According to the alignment between the E5 ORFs of MfuPV1 and the most closely related PV types, the MfuPV1 genome encodes two different E5 proteins, E5-epsilon and E5-zeta. Both novel PVs have a type 1 PDZ-binding motif (xT/SxV/L) at the extreme C-termini of E6 proteins. Furthermore, both E6 proteins contain two conserved zinc-finger domains (CxxC(x)_29_CxxC), separated by 36 amino acids, whereas both E7 proteins exhibit one such domain. In both novel PVs, the E7 protein contains a binding site (LxCxE) for the retinoblastoma tumor suppressor protein (pRB).

At the C-termini of the major (L1) capsid proteins, MfuPV1 and MfuPV2 have two NLSs: the first NLS is comprised of six arginine and lysine residues, whereas the other NLS is bipartite and overlaps with the first NLS (Table 2). In both novel PVs, the minor (L2) capsid protein contains nNLS and cNLS at the extreme N- and C- termini, respectively. The N-termini of both novel L2 proteins contain a consensus furin cleavage motif (RxK/RR) that overlaps with the nNLS and a 23 amino acid residue long transmembrane-like domain consisting of several highly conserved overlapping GxxxG motifs. Interestingly, an additional furin cleavage motif (RRKR) was observed nine amino acid residues upstream of the first motif in the MfuPV1 L2 protein. The central region of the MfuPV1 and MfuPV2 L2 proteins contains an arginine-rich nuclear retention signal (NRS). In addition, a binding site for the L1 protein with a consensus PxxPxxPxxP sequence was identified at the C-terminus of both L2 proteins.

### 3.3. Phylogenetic Analysis of MfuPV1 and MfuPV2 and Sequence Similarities

**Figure 5:**
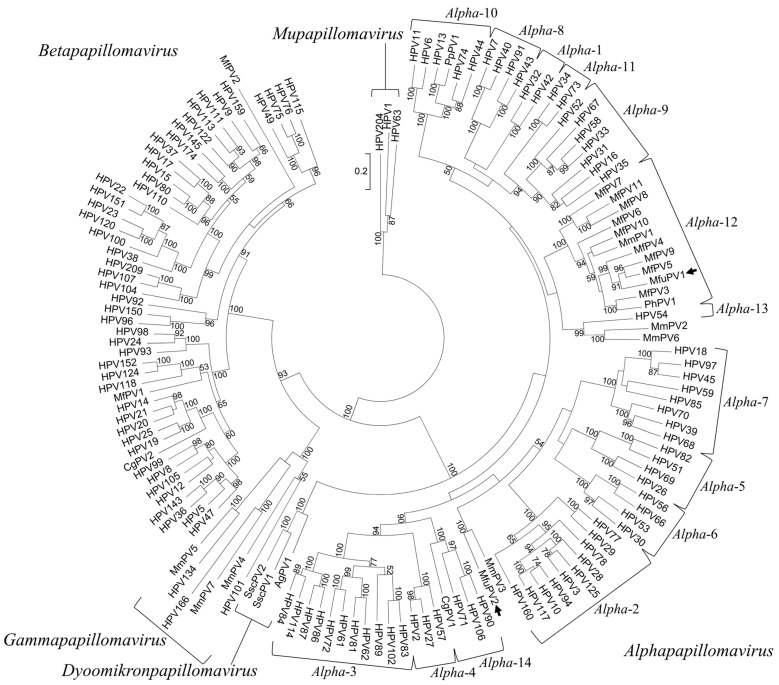
Phylogenetic position of MfuPV1 and MfuPV2. The positions of MfuPV1 and MfuPV2 are indicated by arrows. A maximum likelihood phylogenetic tree was constructed based on the full-length L1 ORF nucleotide sequence alignment of MfuPV1, MfuPV2, and 142 previously characterized papillomavirus (PV) types from the *Alpha*-, *Beta*-, *Gamma*-, *Mu*-, and *Dyoomikron*-PV genera. The nucleotide sequences of the three HPVs forming the *Mu*-PV genus, HPV1, HPV63, and HPV204, were used to root the tree. The numbers at the internal nodes represent bootstrap support values (above 50%), as determined for 1000 iterations by the maximum likelihood method. The evolutionary distances units are number of base substitutions per site.

Based on the initial phylogenetic analysis of the partial L1 ORF nucleotide sequences obtained by the broad-spectrum CODEHOP PCR, the presence of two putative novel viruses belonging to the *Alpha*-PV genus was suggested. In addition, a maximum likelihood phylogenetic tree based on the full-length L1 ORF nucleotide sequence alignment of MfuPV1, MfuPV2, and 142 previously characterized PV types from the *Alpha*-, *Beta*-, *Gamma*-, *Mu*-, and *Dyoomikron*-PV genera, confirmed the results of the preliminary analysis. As shown in Figure 5, MfuPV1 clusters within the viral species *Alpha*-12, closest to MfPV4, MfPV5, and MfPV9, while MfuPV2 is phylogenetically most closely related to MmPV3 and HPV types from the species *Alpha*-2. The similarity of the MfuPV1 L1 ORF nucleotide sequence to the L1 ORFs of the PV types from the viral species *Alpha*-12 ranged from 78.7% to 83.4%, while the identified pairwise L1 nucleotide similarity of MfuPV2 with the phylogenetically most closely related PV type MmPV3 was found to be 77.1% and only 67.8% to 69.7% for *Alpha*-2 species HPV types (Table 3 and Table 4).

The results of additional pairwise nucleotide and amino acid sequence comparisons of the whole genomes, individual viral ORFs, and proteins of MfuPV1 and its most closely related PV types are presented in Table 3. MfuPV1 and MfPV5 showed the highest similarity in the nucleotide and amino acid sequences of the E1, E2, E5-epsilon, E5-zeta, L1, and L2 ORFs and proteins. On the other hand, the MfuPV1 nucleotide and amino acid sequences of the E1^E4 fusion spliced sequence and protein and the E7 ORF were most similar to the corresponding sequences of MfPV9 and MfPV4, respectively. The nucleotide sequence of the MfuPV1 E6 ORF showed the highest similarity to the MfPV9 E6 ORF, while the corresponding MfuPV1 E6 protein sequence was more similar to the MfPV5 E6 protein. The PASC global alignment analysis revealed that MfuPV1 and MfPV5 also share the highest whole genome pairwise similarity (80.5%).

The nucleotide and amino acid sequences of the E1, E2, E6, E7, L1, and L2 ORFs and proteins of MfuPV2 showed the highest similarity to the corresponding ORFs and proteins of MmPV3 (Table 4). Interestingly, the nucleotide sequence of the MfuPV2 E4 ORF was most similar to the HPV77 E4 ORF, while the amino acid sequence of the MfuPV2 putative E4 protein was more similar to the HPV160 putative E4 protein. The nucleotide sequence of the MfuPV2 E5 ORF showed the highest similarity to the HPV29 E5 ORF, while the amino acid sequence of the MfuPV2 E5 protein was more similar to the MmPV3 E5 protein. In addition, the PASC global alignment analysis showed that MfuPV2 has the highest whole genome pairwise similarity with MmPV3 (68.7%).

### 3.4. MfuPV1 and MfuPV2 Viral Load

The MfuPV1 viral load in the oral squamous cell carcinoma tissue of a Japanese macaque was estimated at 711.88 viral DNA copies per single host cell. The MfuPV2 viral load in tissue from the benign penile lesion of a Japanese macaque was estimated at 10.37 viral DNA copies per single host cell (Table 5).

## 4. Discussion

As of 26 February 2021, more than 400 PV types have been completely characterized, from which only 18 have been detected in cynomolgus and rhesus macaques, two species belonging to the same genus as Japanese macaques [6,9,12]. In the present study, we describe the detection of the first two PV types—MfuPV1 and MfuPV2, naturally infecting Japanese macaques. We originally identified MfuPV1 and MfuPV2 in tissue samples of an oral squamous cell carcinoma and a benign penile lesion, respectively, of two Japanese macaques. Molecular analyses revealed that both novel viral genomes exhibit the typical genomic organization of previously described PV types from the *Alpha*-PV genus, including a non-coding LCR region with regulatory sequences and ORFs encoding characteristic proteins with conserved functional domains and motifs enabling viruses to complete their life cycles and infect novel host cells [3]. Both MfuPV1 and MfuPV2 have eight major ORFs, encoding six early (E1, E2, E4, E5, E6, and E7) and two late viral proteins (L1 and L2), where MfuPV1 encodes two different E5 proteins, E5-epsilon and E5-zeta [6,46]. As is typical for PVs, the LCRs of MfuPV1 and MfuPV2 are positioned between the L1 and E6 ORFs and contain at least one putative polyadenylation site for processing late viral mRNA transcripts, four palindromic E2-binding sites, an 18 bp long palindromic E1-binding region, putative binding sites for cellular transcription factors (i.e., C/EBPbeta, YY1, AP-1, NFI/CTF, and SP-1), and, according to the presence of putative TATA box motif, also the major early viral promoter [11,34,43]. According to the published literature [30], the E1-binding region together with the second, third, and fourth E2-binding sites in both LCRs most likely represent the origin of viral DNA replication for these viruses. Between ORFs E2 and E5, both novel viral genomes additionally harbor NCR1s with an unknown function.

The E1 ORFs encode the largest MfuPV1 and MfuPV2 proteins, which together with E2 viral proteins act as origin recognition factors and regulators of viral DNA replication, as well as regulators of early viral transcription in the cellular nucleus [30]. The MfuPV1 and MfuPV2 E1 proteins contain a bipartite NLS and NES within the N-terminal region that may interact with karyopherins, nuclear transport receptors involved in transporting molecules through nuclear pores [47,48]. As is typical for some high-risk HPV types from the *Alpha*-PV genus [34], the downstream NLS region of the MfuPV1 E1 protein consists of nonpolar and polar uncharged amino acids and is therefore most probably not functional. The cyclin-binding motif and cdk-phosphorylation sites are located next to the NLS/NES region, which has the ability to interact with cyclin A/E in a complex with Cdk2. After phosphorylation of the cdk-phosphorylation sites by Cdk2, the binding affinity of the NLS/NES region for karyopherins changes, leading to nuclear accumulation of E1 proteins [3,33,34]. Thus, the N-terminal sequence of E1 provides several motifs that act in concert with cellular factors to regulate E1 protein nucleo-cytoplasmic transport. At the C-termini of both E1 proteins, a conserved ATP-binding site was identified, suggesting that they can act as ATP-dependent DNA helicases [30]. The MfuPV1 and MfuPV2 E2 proteins have a conserved leucine-zipper domain, necessary for dimerization of E2 proteins prior to binding to E2-binding sites in the LCR [30,36], as well as a consensus NLS in the central hinge region, which promotes nuclear localization of the proteins [49].

Both E4 ORFs are characteristically located centrally within the E2 ORFs [37]. While the MfuPV2 E4 ORF contains a start codon, the MfuPV1 E4 ORF lacks one and thus probably can be translated as a fusion protein from the spliced E1^E4 mRNA consisting of the start codon and first few codons of the E1 ORF joined to the E4 ORF [22,30]. Like most HPVs, the MfuPV1 and MfuPV2 E4 proteins contain a consensus “leucine-cluster” motif, which is important for the association between E4 proteins and cytokeratin intermediate filaments, leading to the disruption of the cellular cytokeratin network and to the facilitated virion release from the PV-infected cells in upper epithelial layers [37]. At the extreme C-termini of E6 proteins, MfuPV1 and MfuPV2 have a type 1 PDZ-binding motif, a typical feature of the high-risk *Alpha*-HPV types, which enables interaction with some of the PDZ domain-containing cellular proteins, implicated in the control of cell growth, signaling, and adhesion, leading to their degradation [11,31]. Furthermore, the MfuPV1 and MfuPV2 E6 and E7 proteins contain conserved zinc-finger domains, which seems to be essential for maintaining the structural integrity and formation of multimers of the viral proteins [30]. In both novel PVs, the E7 protein contains a binding site for the pRB and thus might be capable of association with and inactivation of the mentioned protein, which could subsequently lead to the facilitated proliferation of PV-infected cells [30,32].

At the C-termini of the L1 proteins, MfuPV1 and MfuPV2 have two NLSs, promoting the accumulation of L1 proteins in the cellular nucleus where they assemble into capsomeres and then into viral capsids [41,42]. In both novel PVs, the L2 protein contains a binding site for the L1 protein at its C-terminus, which enables the assembly of capsid proteins into capsomeres. In addition, nNLS and cNLS, promoting nuclear localization, were identified at the extreme N- and C- termini of both L2 proteins [38,39]. As described previously [50], a cNLS enables binding of the L2 protein to the cellular motor protein dynein, which interacts with the microtubule network and transfers a complex composed of the L2 protein and viral DNA (L2/vDNA complex) to the cellular nucleus. The MfuPV1 and MfuPV2 L2 proteins contain the furin cleavage motif at their N-termini, which allows their cleavage by the cellular enzyme furin in the cell membrane and causes conformational changes of the viral capsid, leading to internalization of the virions into basal epithelial cells via cell surface entry receptors. Furin cleavage also leads to exposure of the transmembrane-like domain that is important for the vesicular escape of the L2/vDNA complex from the endosomal compartment after cellular uptake [39,40]. The central region of the MfuPV1 and MfuPV2 L2 proteins contains a NRS, which allows for the association of the L2/vDNA complex with the nuclear matrix during the metaphase stage in the cell cycle [38].

Since all data on the molecular features of both novel PVs were obtained by in silico molecular analyses and comparative genomics based on previously well characterized PV types, experimental evidence such as RNA and protein expression as well as mutagenesis studies will be needed to confirm the biological functionality of the described proteins and motifs.

Based on the maximum likelihood phylogenetic tree, MfuPV1 clusters within the viral species *Alpha*-12, closest to MfPV4, MfPV5, and MfPV9, which were identified in the anogenital mucosa of cynomolgus macaques [11]. MfuPV2 is phylogenetically most closely related to MmPV3, isolated from the penile surface of a rhesus macaque [12,51], and to HPV types from the species *Alpha*-2 that are etiologically linked with the development of cutaneous epithelial neoplasms, such as flat and common warts [3,52,53]. Pairwise nucleotide sequence comparisons showed that both MfuPV1 and MfuPV2 have less than 90% L1 ORF nucleotide sequence similarity with phylogenetically most closely related PV types and therefore fulfil the established PV classification criteria for novel PV types. Furthermore, MfuPV1 was identified as a novel representative of the viral species *Alpha*-12. Meanwhile, the MfuPV2 L1 ORF nucleotide sequence differs from *Alpha*-2 species HPV types by more than 30% and thus together with MmPV3 most likely represents a novel viral species within the *Alpha*-PV genus [1]. The results of pairwise sequence comparisons of the whole genomes, other individual viral ORFs, and proteins of specific MfuPV and its most closely related PV types showed that MfPV5, originally detected in a histologically confirmed cervical intraepithelial neoplasia of a cynomolgus macaque [11], is presumably the closest known relative of MfuPV1. On the other hand, it was confirmed that MfuPV2 and MmPV3 are undoubtedly closest relatives to date. The high degrees of nucleotide similarity between the E1, E2, E6, E7, L1, and L2 ORFs of MfuPV2 and MmPV3 (68.8–77.1%), as opposed to the E4 (42.9%) and E5 (50.0%) ORFs, suggest different evolutionary selective pressure and conservation of specific functions of the MfuPV2 E4 and E5 proteins [52,54].

Using newly developed type-specific qPCR assays in combination with the tubulin qPCR [23], viral loads of MfuPV1 and MfuPV2 in the original tissue samples were estimated in order to determine a possible etiological association of the novel PV types with the development of the original neoplasms. The MfuPV1 viral load in the oral squamous cell carcinoma tissue of a Japanese macaque was estimated at 711.88 viral DNA copies per single host cell and was similar to viral load estimates of high-risk HPV16 in samples of oropharyngeal carcinomas, with values ranging from 0.003 to 1080 viral DNA copies per single human cell [55]. Similarly, the MfuPV2 viral load in tissue sample of the benign penile lesion of a Japanese macaque was estimated at 10.37 viral DNA copies per single host cell, which is similar to the low-risk HPV6 viral load in samples of genital warts, with values ranging from 2 to 870 viral DNA copies per single human cell [56]. In addition, based on the estimates of viral load of cutaneous HPV types measured in common skin warts containing a single HPV type and a clear bimodal distribution of the viral loads, the cutoff value for the determination of the causative HPV type was previously set at one HPV DNA copy per single human cell [57]. The comparison of MfuPV1 and MfuPV2 viral loads to viral load data described previously for other *Alpha*-PV types in similar neoplastic lesions, and the detection of only MfuPV1 and MfuPV2 in the original neoplasms using PCR in a combination with broad-spectrum CODEHOP primers strongly suggest a mucosal tissue tropism of both novel PV types and their causative role in the development of these particular neoplasms.

MfuPV1 is the first representative of the species *Alpha*-12 that has been identified in the oral mucosa to date. Nevertheless, there is a possibility that MfuPV1 has the ability to infect both oral and anogenital mucosal epithelia and induce the development of carcinomas, as has been shown for oncogenic HPV16 and HPV18 [58]. The main problem in determining the spectrum of tissue tropism of MfuPVs is that it is difficult to obtain samples of neoplastic lesions from various anatomical regions of Japanese macaques. In addition to MmPV3, MfuPV2 is the second representative of a putatively new viral species within the *Alpha*-PV genus, where both NHP PV types have a mucosal tissue tropism and are related to HPVs with cutaneous tropism. It could be assumed that closely related HPVs with mucosal tissue tropism may also exist in this same viral species, but they have yet to be discovered. Furthermore, close phylogenetic relationship of MfuPV2 and MmPV3 to cutaneous HPVs rather than to other PVs of macaques could be the result of niche adaptation of their ancient PVs and subsequent coevolution with their primate hosts [51]. However, considering that human is the only intensively studied PV host species [4,6], characterization of novel animal PVs, especially NHP PVs that are closely related to clinically important HPV types, remains essential for the understanding of molecular diversity, evolutionary history, and pathogenesis of HPVs [7,11].

## 5. Conclusions

Two novel PVs, MfuPV1 and MfuPV2, initially identified in an oral squamous cell carcinoma and a benign penile lesion of Japanese macaques, respectively, are the only known PV types infecting this primate species to date. Therefore, the identification and characterization of MfuPV1 and MfuPV2 improves our knowledge of the molecular diversity and evolutionary history of NHP PVs. The detailed in silico molecular analyses revealed that both novel PV genomes exhibit the typical genomic organization of *Alpha*-PV types, including a non-coding genomic region LCR with regulatory sequences and ORFs encoding characteristic proteins with conserved functional domains and motifs to regulate and complete the viral life cycle. However, additional experimental evidence is needed to finally confirm the presence and biological functionality of the molecular features of both novel PVs. Phylogenetically, MfuPV1 clusters within the viral species *Alpha*-12 and is most closely related to PVs that have been previously identified in the anogenital mucosa of cynomolgus and rhesus macaques (MfPV3–11 and MmPV1). MfuPV2, together with MmPV3, is most likely a representative of a novel viral species within the *Alpha*-PV genus and beyond that is closely related to HPV types from the species *Alpha*-2, which are associated with cutaneous epithelial neoplasms. Based on the relatively high MfuPV1 and MfuPV2 viral load estimates and the fact that no other PV types besides the novel *Alpha*-PV types were detected in the tissue lesions, it could be assumed with high certainty that MfuPV1 and MfuPV2 exhibit a mucosal tissue tropism and are etiologically linked with the development of the original neoplastic lesions.

## Figures and Tables

**Table 1:** All currently recognized papillomavirus types in different species of macaques (genus *Macaca*).

Macaque Species (Genus *Macaca*)	PV Type	PV Genus	Sample Origin	Reference
cynomolgus macaque (*M. fascicularis*)	MfPV1, -2	*Beta*-PV	palmoplantar skin papillomas	[8,10]
	MfPV3, -4, -5, -8	*Alpha*-PV	cervical intraepithelial neoplasia	[11]
	MfPV6, -7, -9, -10, -11	*Alpha*-PV	exfoliated cervical cytology samples	[11]
rhesus macaque (*M. mulatta*)	MmPV1	*Alpha*-PV	lymph node metastasis of a penile squamous cell carcinoma	[13]
	MmPV2	*Alpha*-PV	exfoliated vaginal cells	[12]
	MmPV3, -6	*Alpha*-PV	swabs of the penile surface	[12]
	MmPV4, -5, -7	*Gamma*-PV	swabs of the penile surface	[12]
Japanese macaque (*M. fuscata*)	MfuPV1	*Alpha*-PV	oral squamous cell carcinoma	this study
	MfuPV2	*Alpha*-PV	benign penile lesion	this study

PV—papillomavirus; MfPV—Macaca fascicularis papillomavirus; MmPV—Macaca mulatta papillomavirus; MfuPV—Macaca fuscata papillomavirus.

**Table 2:** Characteristics of MfuPV1 and MfuPV2 putative motifs and domains.

Genomic Region	Motifs and Domains (Consensus Sequence)	MfuPV1	MfuPV2
E6	Zinc-finger domain (CxxC(x)_29_CxxC) [30]	aa 30–66;	aa 29–65;
		aa 103–139	aa 102–138
	PDZ-binding motif (xT/SxV/L) [31]	aa 148–151	aa 145–148
E7	pRB-binding site (LxCxE) [32]	aa 22–26	aa 23–27
	Zinc-finger domain (CxxC(x)_29_CxxC) [30]	aa 58–94	aa 69–105
E1	Bipartite NLS [33,34]	aa 85–87…120–123 ^a^	aa 93–95…128–131
	NES (Lx_(2–3)_Lx_2_(L/I/V)x(L/I)) [34]	aa 106–115	aa 114–123
	Cyclin-binding motif (RxL) [33,34]	aa 124–126	aa 132–134
	Cdk-phosphorylation site ((S/T)P) [33]	aa 91–92;	aa 99–100;
		aa 95–96;	aa 103–104;
		aa 107–108	aa 115–116
	ATP-binding site (Gx_4_GK(T/S)) [34]	aa 457–464	aa 482–489
E2	NLS (KRxR) [8,35]	aa 242–245	aa 244–247
	Leucine-zipper domain (Lx_6_Lx_6_Lx_6_L) [36]	aa 287–308	aa 306–327
E1^E4/E4	“Leucine-cluster” motif (LLxLL) [37]	aa 14–18	aa 60–64 ^a^
	Polyadenylation site (AATAAA) [11]	nt 4224–4229	nt 4352–4357
L2	nNLS [38]	aa 1–11	aa 1–11
	cNLS [38]	aa 455–460	aa 448–452
	Furin cleavage motif (RxK/RR) [39]	aa 8–11;	aa 8–11;
		aa 20–23	absent
	Transmembrane-like domain (GxxxG motifs) [40]	aa 56–78	aa 44–66
	NRS [38]	aa 303–323	aa 292–312
	L1-binding domain (PxxPxxPxxP) [39]	aa 425–434	aa 414–423
L1	Monopartite NLS [41,42]	aa 494–499	aa 500–505
	Bipartite NLS [41,42]	aa 481–482...494–495	aa 487–488...500–501
LCR	Polyadenylation site (AATAAA) [11,20]	nt 7273–7278	nt 7405–7410
	Polyadenylation site (ATTAAA) [20]	absent	nt 7444–7449
	E2-binding site (ACC(N)_6_GGT) [30]	nt 7436–7447;	nt 7606–7617;
		nt 7752–7763;	nt 7915–7926;
		nt 7829–7840;	nt 7994–8005;
		nt 7844–7855	nt 8009–8020 ^a^
	E1-binding region [8,34]	nt 7789–7806	nt 7954–7971
	TATA box (TATAAA) [43]	nt 7859–7864	nt 8024–8029
	C/EBPbeta-binding site (T(T/G)NNGNAA(T/G)) [43,44]	nt 7189–7197 ^a^	nt 7780–7788
	YY1-binding site (CCGCCATNTT) [43]	nt 7398–7408 ^a^	absent
	AP-1-binding site (TGANTCA) [43]	nt 7556–7562	nt 7650–7656
	NFI/CTF-binding site (TTGGC) [43]	nt 7624–7628;	nt 7161–7165;
		nt 7667–7671	nt 7719–7723
	SP-1-binding site (NGGNGN) [43,45]	nt 7822–7827	nt 7987–7992

^a^ Sequence that differs from the consensus sequence; MfuPV—Macaca fuscata papillomavirus; nt—nucleotide; aa—amino acid; pRB—retinoblastoma tumor suppressor protein; NLS—nuclear localization signal; NES—nuclear export signal; NRS—nuclear retention signal; Cdk—cyclin-dependent kinase.

**Table 3:** Nucleotide and amino acid sequence similarities between whole genomes, individual viral open reading frames (ORFs), and proteins (E6, E7, E1, E2, E1^E4, E5-epsilon, E5-zeta, L2, and L1) of MfuPV1 and most closely related papillomavirus types.

Genomic Region	Pairwise Similarity with MfuPV1 ^a^ (%)	MfPV5 ^a^	MfPV9 ^a^	MfPV4 ^a^	MmPV1 ^a^	MfPV10 ^a^
E6	nucleotide sequence	80.3	80.5	78.1	64.9 ^b^	75.7
	amino acid sequence	80.3	79.6	80.3	63.6	73.7
E7	nucleotide sequence	72.0 ^b^	71.5	75.0	66.1	69.9
	amino acid sequence	58.9	68.0	68.0	57.9	65.4
E1	nucleotide sequence	86.0	85.4	80.4	79.2	79.6
	amino acid sequence	88.9	88.7	82.1	82.0	82.9
E2	nucleotide sequence	79.6	79.1	77.4 ^b^	72.7	76.9
	amino acid sequence	73.4	72.1	72.1	64.8	69.9
E1^E4	nucleotide sequence	72.7	75.4	69.5	69.7	68.9
	amino acid sequence	62.2	65.9	54.7	52.3	53.8
E5-epsilon	nucleotide sequence	85.7	82.5	78.0	67.4	74.4
	amino acid sequence	85.7	85.7	72.7	69.8	76.7
E5-zeta	nucleotide sequence	80.1	76.7	68.6 ^c^	64.6	68.8
	amino acid sequence	74.5	74.0	54.9	56.3	53.2
L2	nucleotide sequence	79.2	77.7	74.1	72.8	73.6
	amino acid sequence	84.5	80.7	77.5	75.9	78.5
L1	nucleotide sequence	83.4	82.9	79.3	79.0	78.7
	amino acid sequence	90.2	90.2	87.9	85.7	88.4
whole genome	nucleotide sequence	80.5	78.7	76.6	72.5	75.7
	amino acid sequence	/	/	/	/	/

^a^ PV types exhibit a mucosal tissue tropism [11,13]; ^b^ ORFs are not equal in length—percentage similarity was calculated from the first common ATG of the two PV types; ^c^ ORFs are not equal in length and without common ATG—percentage similarity of the full-length ORF sequences was calculated; PV—papillomavirus; MfuPV—Macaca fuscata papillomavirus; MfPV—Macaca fascicularis papillomavirus; MmPV—Macaca mulatta papillomavirus; ORF—open reading frame.

**Table 4:** Nucleotide and amino acid sequence similarities between whole genomes, individual viral open reading frames (ORFs), and proteins (E6, E7, E1, E2, E4, E5, L2, and L1) of MfuPV2 and most closely related papillomavirus types.

Genomic Region	Pairwise Similarity with MfuPV2 ^a^ (%)	MmPV3 ^a^	HPV160 ^b^	HPV117 ^b^	HPV77 ^b^	HPV29 ^b^
E6	nucleotide sequence	69.8	56.4	56.6	57.4	58.5
	amino acid sequence	62.4	46.3	46.3	48.3	49.0
E7	nucleotide sequence	68.8	58.3	43.6 ^c^	59.8	59.8
	amino acid sequence	67.0	52.3	44.9	56.1	56.1
E1	nucleotide sequence	73.7	67.7	65.7	69.1	68.6
	amino acid sequence	75.0	66.7	65.4	68.7	66.6
E2	nucleotide sequence	69.3	63.6	61.9	62.8	61.1
	amino acid sequence	60.5	56.4	51.7	54.8	54.9
E4	nucleotide sequence	42.9 ^c^	54.0 ^d^	- ^e^	56.1 ^d^	40.8 ^c^
	amino acid sequence	33.3	35.2	-	34.3	19.6
E5	nucleotide sequence	50.0 ^f^	47.6 ^c^	47.2 ^c^	48.6 ^d^	51.4 ^d^
	amino acid sequence	42.6	26.5	20.4	38.5	22.2
L2	nucleotide sequence	70.4	63.7	63.7	63.4	65.4
	amino acid sequence	73.2	63.9	63.7	63.2	64.6
L1	nucleotide sequence	77.1	69.7	68.4	68.1	67.8
	amino acid sequence	82.9	72.0	72.0	72.4	71.0
whole genome	nucleotide sequence	68.7	58.5	57.1	58.4	58.8
	amino acid sequence	/	/	/	/	/

^a^ PV types exhibit a mucosal tissue tropism [12]; ^b^ PV types exhibit a cutaneous tissue tropism [3]; ^c^ ORFs are not equal in length and without common ATG—percentage similarity of the full-length ORF sequences was calculated; ^d^ ORFs are not equal in length—percentage similarity was calculated from the first common ATG of the two PV types; ^e^ ORF lacks its own start codon; ^f^ The ORF was determined using the ORF finder web-based service; PV—papillomavirus; MfuPV—Macaca fuscata papillomavirus; MmPV—Macaca mulatta papillomavirus; HPV—human papillomavirus; ORF—open reading frame.

**Table 5:** Concentrations of viral DNA, genomic DNA, and viral load in MfuPV1- and MfuPV2-positive tissue samples of neoplasms of Japanese macaques (*Macaca fuscata*).

PV Type	Sample	Concentration of Viral DNA (Copies/µL)	Concentration of Genomic DNA (ng/µL)	Concentration of Genomic DNA (Cell/µL)	Viral Load (Copies/Cell)
MfuPV1	oral squamous cell carcinoma	1.013 × 10^6^	10.133	1.423 × 10^3^	711.876
MfuPV2	benign penile lesion	3.04 × 10^4^	20.867	2.931 × 10^3^	10.372

PV—papillomavirus; MfuPV—Macaca fuscata papillomavirus.

## Data Availability

The authors confirm that the data supporting the findings of this study are available within the article and are openly available in the GenBank database at https://www.ncbi.nlm.nih.gov/genbank/ under accession numbers KT944080 and MH469677.

## Supplementary Materials

The following are available online at https://www.mdpi.com/article/10.3390/v13040630/s1, Table S1: Primers used for MfuPV1 whole genome sequencing using a primer walking strategy, Table S2: Primers used for MfuPV2 whole genome sequencing using a primer walking strategy, Table S3: Accession numbers of papillomavirus types used to perform the phylogenetic and sequence similarity analyses.
