# Supplementary material for: Molecular and Phylogenetic Characterization of Novel Papillomaviruses Isolated from Oral and Anogenital Neoplasms of Japanese Macaques (Macaca fuscata)
Source: Viruses. 2021 Apr 7;13(4):630. doi: 10.3390/v13040630 (PMC8067741; doi:10.3390/v13040630)
Supplement: Supplementary file 1 [file viruses-13-00630-s001.pdf]

Supplementary Materials

Table S1: Primers used for MfuPV1 whole genome sequencing using a primer walking strategy.

Primer name	Orientation	Sequence (5'-3')
MfuPV1-M13f4A	Forward	GGCAAAGTGTAACTGAAATGG
MfuPV1-M13f7A	Forward	ACTCTATATGAAGATGATAGCAAAGA
MfuPV1-M13f7B	Forward	AAGGACAATGGAATGGACTACA
MfuPV1-M13f7C	Forward	AGGGCATTCCTGTGACC
MfuPV1-M13f7D	Forward	GCCCTCCCGTATCAGTG
MfuPV1-M13f7E	Forward	CGTGCCACTCAGCAGGTT
MfuPV1-M13f7F	Forward	GTCCTTGCTCCACCACAG
MfuPV1-M13f7G	Forward	CTTGCACAGCTAATCCCTCT
MfuPV1-M13r1A	Reverse	CTTTCCTGGTAGCCCTCC
M13 Reverse.1[a]	Reverse	GAAACCTTGGGAACAGAC
M13 Forward.1[a]	Forward	CCTTGGAGGACACCTATC
M13 Reverse.2[a]	Reverse	TGACCTAGTCGGCTAAGC
M13 Forward.2[a]	Forward	CCTTGCCGAATACTCTGC
M13 Reverse.3[a]	Reverse	TGCCAGCCTCAATAAGAC
M13 Forward.3[a]	Forward	GTGTCAACTGTGCTTAAG
M13 Reverse.4[a]	Reverse	CATTCTGCTGTTCACTAC
M13 Forward.4[a]	Forward	AGGACCTTCGTGTGTTAG
M13 Reverse.5[a]	Reverse	ACAAAGCCACGTTACTTG
M13 Forward.5[a]	Forward	CCGCCTAAGCTGCTTTTG
M13 Reverse.6[a]	Reverse	TTTGTCCAATGCCAGGTG
M13 Forward.6[a]	Forward	ACAGTGGATAAAACGTCG
M13 Reverse.7[a]	Reverse	ATCTCGCAGTGTCCACTC
M13 Forward.7[a]	Forward	TTAAGTGCGCTGCAGGAC

[a] Primers were designed at Microsynth AG (Balgach, Switzerland).

Table S2: Primers used for MfuPV2 whole genome sequencing using a primer walking strategy.

Primer name	Orientation	Sequence (5'-3')
MfuPV2-s1-forward	Forward	AAAATCCAAACGCTAAAATC
MfuPV2-s2-forward	Forward	TGCTTTTAAGGAACATTTTTT
MfuPV2-s3-forward	Forward	ATTATACCTGGTGTGGAAGAA
MfuPV2-s4-forward	Forward	GAGGCAGTGGTTACTAAGAGG
MfuPV2-s5-forward	Forward	GTAAGTTTCAGGACCTTTTTG
MfuPV2-s6-forward	Forward	GCAGCATTATATTGGTACAGG
MfuPV2-s7-forward	Forward	TGTCAATGTCAGAGTGGATAA
MfuPV2-s8-reverse	Reverse	TCGCTTAACTTGTTGCTATCT
MfuPV2-sDOD-forward	Forward	CAGGATCAAATGCTAGAACTG
MfuPV2-s9-forward	Forward	GACAAAAGTAAAGGGACAAAT
MfuPV2-s10-forward	Forward	GACAGTTCACCTGTAATCCAC
MfuPV2-s1011-forward	Forward	GTTTATATCTGGAGGTGCTGT
MfuPV2-s11-forward	Forward	TACTGTTGCTGGTGTTATTGA
MfuPV2-s12-forward	Forward	TTTAGCAACCCTCTGTTTACT
MfuPV2-s13-forward	Forward	CGTGTATGTTGATGGTAGTGA
MfuPV2-249f1	Forward	CAATCAAGGGGACACAGTCC
MfuPV2-145r1	Reverse	TGCTCACGCCTCAGATAAAA
V2_r.2[a]	Reverse	TTGATCGGTGCTTACAAC
V2_r.3[a]	Reverse	TGCAGTGAGACAATATCC
V2_r.4[a]	Reverse	TAACATCCACTACAGTGC
V2_r.5[a]	Reverse	AGAATTAGGAAGCAGATG
V2_r.6[a]	Reverse	TCGCTGTCGTTTAGGTAC
V2_r.7[a]	Reverse	TGTTTCATACAGTTCTAG
V2_f.2[a]	Forward	TCCACTCCCGCTGCCAAG
V2_f.3[a]	Forward	AAGTGCAACTCTGGCAAC
V2_f.4[a]	Forward	AATTGAGGCAGAAACCGG
V2_f.5[a]	Forward	ACATGGTGGACTTCATAG
V2_f.6[a]	Forward	GGTGACATCTTGCAACTG
V2_f.7[a]	Forward	TTTTGCATGAGCCTTATG
MfuPV2-E1-invforward	Forward	GGACACAGTGCTGAAAACTCT
MfuPV2-E1-invreverse	Reverse	CTGTTCTTCGCACATTTGAA
pJET1.2 Forward[b]	Forward	CGACTCACTATAGGGAGAGCGGC
pJET1.2 Reverse[b]	Reverse	AAGAACATCGATTTTCCATGGCAG

[a] Primers were designed at Microsynth AG (Balgach, Switzerland).

[b] Primers are included in CloneJET PCR Cloning Kit (Thermo Scientific, Waltham, MA, USA).

Table S3: Accession numbers of papillomavirus types used to perform the phylogenetic and sequence similarity analyses.

PV type	PV genus	GenBank[a] accession number	PV type	PV genus	GenBank[a] accession number
HPV1	*Mu*-PV	V01116	HPV43	*Alpha*-PV	AJ620205
HPV2	*Alpha*-PV	X55964	HPV44	*Alpha*-PV	U31788
HPV3	*Alpha*-PV	X74462	HPV45	*Alpha*-PV	X74479
HPV5	*Beta*-PV	M17463	HPV47	*Beta*-PV	M32305
HPV6	*Alpha*-PV	X00203	HPV49	*Beta*-PV	X74480
HPV7	*Alpha*-PV	X74463	HPV51	*Alpha*-PV	M62877
HPV8	*Beta*-PV	M12737	HPV52	*Alpha*-PV	X74481
HPV9	*Beta*-PV	X74464	HPV53	*Alpha*-PV	X74482
HPV10	*Alpha*-PV	X74465	HPV54	*Alpha*-PV	U37488
HPV11	*Alpha*-PV	M14119	HPV56	*Alpha*-PV	X74483
HPV12	*Beta*-PV	X74466	HPV57	*Alpha*-PV	X55965
HPV13	*Alpha*-PV	X62843	HPV58	*Alpha*-PV	D90400
HPV14	*Beta*-PV	X74467	HPV59	*Alpha*-PV	X77858
HPV15	*Beta*-PV	X74468	HPV61	*Alpha*-PV	U31793
HPV16	*Alpha*-PV	K02718	HPV62	*Alpha*-PV	AY395706
HPV17	*Beta*-PV	X74469	HPV63	*Mu*-PV	X70828
HPV18	*Alpha*-PV	X05015	HPV66	*Alpha*-PV	U31794
HPV19	*Beta*-PV	X74470	HPV67	*Alpha*-PV	D21208
HPV20	Beta-PV	U31778	HPV68	*Alpha*-PV	DQ080079
HPV21	Beta-PV	U31779	HPV69	*Alpha*-PV	AB027020
HPV22	*Beta*-PV	U31780	HPV70	*Alpha*-PV	U21941
HPV23	*Beta*-PV	U31781	HPV71	*Alpha*-PV	AB040456
HPV24	*Beta*-PV	U31782	HPV72	*Alpha*-PV	X94164
HPV25	*Beta*-PV	X74471	HPV73	*Alpha*-PV	X94165
HPV26	*Alpha*-PV	X74472	HPV74	*Alpha*-PV	AF436130
HPV27	*Alpha*-PV	X74473	HPV75	*Beta*-PV	Y15173
HPV28	*Alpha*-PV	U31783	HPV76	*Beta*-PV	Y15174
HPV29	*Alpha*-PV	U31784	HPV77	*Alpha*-PV	Y15175
HPV30	*Alpha*-PV	X74474	HPV78	*Alpha*-PV	KC138720
HPV31	*Alpha*-PV	J04353	HPV80	*Beta*-PV	Y15176
HPV32	*Alpha*-PV	X74475	HPV81	*Alpha*-PV	AJ620209
HPV33	*Alpha*-PV	M12732	HPV82	*Alpha*-PV	AB027021
HPV34	*Alpha*-PV	X74476	HPV83	*Alpha*-PV	AF151983
HPV35	*Alpha*-PV	X74477	HPV84	*Alpha*-PV	AF293960
HPV36	*Beta*-PV	U31785	HPV85	*Alpha*-PV	AF131950
HPV37	*Beta*-PV	U31786	HPV86	*Alpha*-PV	AF349909
HPV38	*Beta*-PV	U31787	HPV87	*Alpha*-PV	AJ400628
HPV39	*Alpha*-PV	M62849	HPV89	*Alpha*-PV	AF436128
HPV40	*Alpha*-PV	X74478	HPV90	*Alpha*-PV	AY057438
HPV42	*Alpha*-PV	M73236	HPV91	*Alpha*-PV	AF419318

Table S3: *Cont.*

PV type	PV genus	GenBank[a] accession number	PV type	PV genus	GenBank[a] accession number
HPV92	*Beta*-PV	AF531420	HPV160	*Alpha*-PV	AB745694
HPV93	*Beta*-PV	AY382778	HPV166	*Gamma*-PV	JX413104
HPV94	*Alpha*-PV	AJ620211	HPV174	*Beta*-PV	HF930491
HPV96	*Beta*-PV	AY382779	HPV204	*Mu*-PV	KP769769
HPV97	*Alpha*-PV	DQ080080	HPV209	*Beta*-PV	KY242583
HPV98	*Beta*-PV	FM955837	AgPV1	*Dyoomikron*-PV	KP861980
HPV99	*Beta*-PV	FM955838	CgPV1	*Alpha*-PV	GU014532
HPV100	*Beta*-PV	FM955839	CgPV2	*Beta*-PV	GU014533
HPV101	*Gamma*-PV	DQ080081	MfPV1	*Beta*-PV	EF028290
HPV102	*Alpha*-PV	DQ080083	MfPV2	*Beta*-PV	GU014531
HPV104	*Beta*-PV	FM955840	MfPV3	*Alpha*-PV	EF558839
HPV105	*Beta*-PV	FM955841	MfPV4	*Alpha*-PV	EF558841
HPV106	*Alpha*-PV	DQ080082	MfPV5	*Alpha*-PV	EF558843
HPV107	*Beta*-PV	EF422221	MfPV6	*Alpha*-PV	EF558840
HPV110	*Beta*-PV	EU410348	MfPV7	*Alpha*-PV	EF558838
HPV111	*Beta*-PV	EU410349	MfPV8	*Alpha*-PV	EF558842
HPV113	*Beta*-PV	FM955842	MfPV9	*Alpha*-PV	EU490516
HPV114	*Alpha*-PV	GQ244463	MfPV10	*Alpha*-PV	EU490515
HPV115	*Beta*-PV	FJ947080	MfPV11	*Alpha*-PV	GQ227670
HPV117	*Alpha*-PV	GQ246950	MmPV1	*Alpha*-PV	M60184
HPV118	*Beta*-PV	GQ246951	MmPV2	*Alpha*-PV	MG837557
HPV120	*Beta*-PV	GQ845442	MmPV3	*Alpha*-PV	MG837558
HPV122	*Beta*-PV	GQ845444	MmPV4	*Gamma*-PV	MG837559
HPV124	*Beta*-PV	GQ845446	MmPV5	*Gamma*-PV	MH745747
HPV125	*Alpha*-PV	FN547152	MmPV6	*Alpha*-PV	MH745748
HPV134	*Gamma*-PV	GU117634	MmPV7	*Gamma*-PV	MH745749
HPV143	*Beta*-PV	HM999995	PhPV1	*Alpha-PV*	JF304764
HPV145	*Beta*-PV	HM999997	PpPV1	*Alpha-PV*	X62844
HPV150	*Beta*-PV	FN677755	SscPV1	*Dyoomikron*-PV	JF304765
HPV151	*Beta*-PV	FN677756	SscPV2	*Dyoomikron*-PV	JF304766
HPV152	*Beta*-PV	JF304768	**MfuPV1**	***Alpha*-PV**	**KT944080**
HPV159	*Beta*-PV	HE963025	**MfuPV2**	***Alpha*-PV**	**MH469677**

[a] GenBank database (NCBI) is available at https://www.ncbi.nlm.nih.gov/genbank/.
PV – papillomavirus; HPV – human papillomavirus; AgPV – Alouatta guariba papillomavirus; CgPV – Colobus guereza papillomavirus; MfPV – Macaca fascicularis papillomavirus; MmPV – Macaca mulatta papillomavirus; PhPV – Papio hamadryas papillomavirus; PpPV – Pan paniscus papillomavirus; SscPV – Saimiri sciureus papillomavirus; MfuPV – Macaca fuscata papillomavirus
